# Inhibition of *de novo* fatty acid synthesis in *Mycobacterium tuberculosis*

**DOI:** 10.1016/j.jbc.2025.111022

**Published:** 2025-12-08

**Authors:** Emma K. Roszkowski, Sarita Charap, Christine R. Montague, Paridhi Sukheja, Case W. McNamara, Paul S. Soma, M Nurul Islam, Barbara Graham, Anna E. Grzegorzewicz, Mary Jackson, Baiyuan Yang, Anthony G. Hay, David G. Russell, John T. Belisle, Brian C. VanderVen

**Affiliations:** 1 Microbiology & Immunology, Cornell University, Ithaca, New York, USA; 2 Calibr-Skaggs Institue for Innovative Medicines, a Division of The Scripps Research Institute, La Jolla, California, USA; 3 Department of Microbiology, Immunology and Pathology, Colorado State University, Fort Collins, Colorado, USA; 4 Department of Chemistry, Biochemistry and Physics, South Dakota State University, Brookings, South Dakota, USA; 5 Department of Microbiology, Cornell University, Ithaca, New York, USA; 6 Mycobacteria Research Laboratories, Department of Microbiology, Immunology and Pathology, Colorado State University, Fort Collins, Colorado, USA

**Keywords:** Fas1, Fas2, fatty acid synthase, *de novo* fatty acid synthesis, Tuberculosis, Mycobacterium, lipid synthesis, drug development

## Abstract

*De novo* fatty acid synthesis produces the acyl units needed to generate phospholipids, lipoproteins, enzyme prosthetic factors, polyketides, and mycolic acids in *mycobacterium tuberculosis* (Mtb). Here, we identified sALT629, a butoxyphenyl-tetrazole-acetamide compound that inhibits *de novo* lipid synthesis in Mtb. This compound disrupts the Mtb lipidome and prevents incorporation of metabolic tracers into acyl chains of Mtb lipids. Unexpectedly, we also found that sALT629 treatment significantly depleted triacylglycerol (TAG) pools as a metabolic compensation mechanism when *de novo* fatty acid synthesis was inhibited. Resistance to sALT629 was mediated by loss of function mutations in HadC, the non-essential hydroxyacyl- acyl carrier protein -dehydratase subunit involved in the synthesis of long-chain oxygenated mycolic acids. Inactivating HadC rescued sALT629-mediated inhibition by sustaining TAG pools to fulfill Mtb’s biosynthetic demand for acyl chains. Lastly, loss of function HadC resistance mutations resulted in cell wall perturbations that confer fitness defects *in vitro* and *in vivo* suggesting that this specific resistance mechanism is unlikely to arise in Mtb in a clinical setting. Significance. Having effective antibiotics to treat tuberculosis underpins our ability to control this disease. The spread of antibiotic-resistant tuberculosis has prompted a need to identify new drug candidates with new mechanisms of action. Here we describe an antitubercular that targets *de novo* fatty acid synthesis in Mtb, a critical process required to generate multiple essential lipid and lipid-based factors in the bacteria. One resistance mechanism to this inhibitor is associated with perturbations to mycolic acid synthesis resulting in Mtb attenuation. These findings demonstrate that *de novo* fatty acid synthesis in Mtb is an actionable drug target, and uncovered a compensatory metabolic resistance network between TAGs and mycolic acids.

*Mycobacterium tuberculosis* (Mtb), the causative agent of tuberculosis (TB), infects over 10 million new individuals and kills more than 1 million people each year (1). Additionally, the ∼400,000 new cases of multidrug resistant TB reported annually underscore the pressing need to discover the next generation of TB drugs with the potential to shorten treatment duration, increase cure rates, and circumvent existing clinical resistance.

Mtb is surrounded by a complex, lipid-rich cell envelope that provides protection against killing by antibiotics and the immune system. In Mtb, *de novo* fatty acid synthesis produces the acyl units needed to generate phospholipids (2), lipoproteins (3), lipoic acid (4), biotin (5), mycobactin (6), and other polyketide lipids (7). These lipid products play key roles in stabilizing the bacterial cell membrane, acquiring iron, supporting enzyme function, and promoting Mtb virulence making lipid synthesis in this pathogen a desirable drug target. In Mtb, the synthesis of the ∼C_14_-C_26_ acyl units occurs *via* successive elongation reactions catalyzed by the fatty acid synthase-1 (Fas1) enzyme using malonyl- coenzyme A (CoA) precursors (8). These acyl chains can be transferred to glycerol-3-phosphate generating phosphatidic acid which is modified to cytidine-diphosphate-diacylglycerol, a central precursor to Mtb’s phospholipids (9, 10, 11, 12, 13, 14). Removal of a phosphate from phosphatidic acid produces diacylglycerol (DAG) that can be attached to Mtb’s lipoproteins (3) or acylated a third time to generate triacylglycerol (TAG) (15). Moreover, Mtb adenylates Fas1 derived fatty acids for loading on acyl carrier protein (ACP) domains of polyketide synthase enzymes to serve as primers for polyketide formation (16). Fas1-derived ∼ C_14_-C_26_ acyl units also serve both as substrates and primers for the synthesis of mycolic acids, the major α-alkyl, β-hydroxy fatty acids that comprise the Mtb cell wall (17). To produce mycolic acids, the Fas1-derived ∼ C_14_-C_26_ acyl units are elongated to a ∼C_60_ mero-mycolate by the fatty acid synthase-2 (Fas2) complex (18). An additional, Fas1-derived ∼ C_26_ is condensed with the mero-mycolate to form the α-chain of mature mycolic acids (17, 19). Shorter acyl chains are also needed to synthesize key prosthetic cofactors in Mtb. Octanoic acid is used for lipoic acid synthesis, an essential cofactor of the pyruvate dehydrogenase, peroxynitrite reductase/peroxidase, and branched-chain ketoacid dehydrogenase enzymes (4, 20). It is thought that two rounds of Fas1-mediated acyl chain elongation generate the precursors needed to produce pimeloyl-ACP-methyl ester, a product required for *de novo* biotin biosynthesis (5, 21). Thus, inhibiting *de novo* fatty acid synthesis in Mtb is predicted to block the production of multiple essential species required for critical processes in the bacterium.

The process of *de novo* fatty acid synthesis in Mtb also requires several ancillary reactions and pathways highlighting the vast drug target space in this pathway. Specifically, the ACP domains of Fas1 and AcpM of Fas2 are activated by the addition of 4′-phosphatepantetheinyl (P-pant) *via* phosphopantetheinyl transferases (PPTases) (22). Thus, loss or inhibition of specific PPTases can prevent fatty acid formation or elongation *via* Fas1 and Fas2 (23, 24). Additionally, Mtb must synthesize malonyl-CoA, the main extender unit for fatty acid biosynthesis which is generated *via* the biotin-dependent acetyl-CoA-carboxylase complex (ACC) that carboxylates acetyl-CoA to form malonyl-CoA (25). Synthesis of CoA requires α-ketoisovalerate and L-aspartate (26), substrates that are also critical metabolic cofactors and the source of P-pant (26). Disrupting any of these ancillary reactions is predicted to inhibit *de novo* fatty acid synthesis in Mtb.

In this study, metabolic tracing and characterization of the Mtb lipidome in response to compound was used to show that sALT629 inhibits *de novo* fatty acid synthesis. Inhibiting *de novo* fatty acid synthesis blocks production of critical structural lipids such as phospholipids and mycolic acids in Mtb. We also demonstrate that loss of function mutations in HadC compromise Fas2 activity and this confers resistance to sALT629 by increasing TAG pools which are likely salvaged to sustain lipid synthesis in the presence of sALT629. This study highlights how inhibiting *de novo* synthesis in Mtb impacts lipid biosynthetic pathways, provides a framework for identifying future inhibitors with this mechanism of action, and describes a drug target in Mtb with a new mechanism of resistance.

## Results

### sALT629 activity and the transcriptional response in Mtb

**Figure 1:**
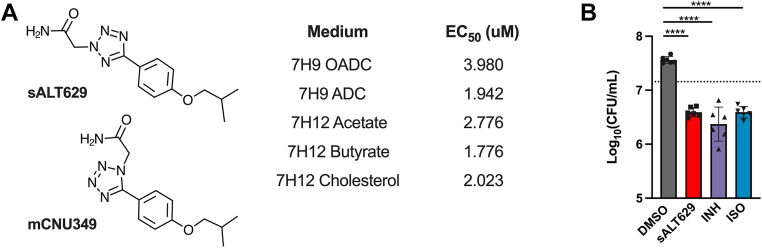
**Inhibitory activity of sALT629.***A*, structure of sALT629 and its activity against WT Mtb in the indicated media. EC_50_ = the concentration of sALT629 to achieve half-maximal inhibitory activity. Values are from two biological replicates with three technical replicates each (n = 6). The analog of sALT629 (mCNU349) was used to isolate spontaneous mutants with cross-resistance to sALT629. *B*, inhibitory activity of sALT629 was evaluated with WT Mtb in 7H12 butyrate media containing the vehicle control (DMSO) or inhibitors (50 μM sALT629, 25 μM INH, or 25 μM ISO) and CFUs were quantified after 5 days of exposure. *Graph* represents data from three biological replicates with two technical replicates each (n = 6). *Horizontal line* indicates bacterial numbers at Day 0 of the assay. *Error bars* represent the SD and data were analyzed using one-way ANOVA with Dunnett’s multiple comparisons test (∗∗∗∗*p* < 0.0001).

We identified sALT629 in a screen for chemical entities that inhibit Mtb growth in macrophages. In this screen, ∼1.1 million compounds were evaluated against macrophages infected with a Mtb strain that constitutively expressed mCherry as a surrogate marker for intracellular growth (27). Compounds that reduced the mCherry signal >10-fold relative to the dimethyl sulfoxide (DMSO) control were selected in this screen. Hit compounds emerging from macrophage-based screens against intracellular Mtb can display conditional inhibitory activities dependent on the carbon source (28). Thus, we evaluated the inhibitory activity of sALT629 in different media formulations. We found that sALT629 inhibits Mtb growth both in macrophages and in medium containing various carbon sources, including host-relevant lipids, with EC_50_ values ranging from 1.8 to 4 μM and sALT629 has an inhibitory profile equivalent to isoniazid (INH) and isoxyl (ISO) following 5-days exposure to the compound (Fig. 1).

In attempt to group sALT629 with compounds that have well-established mechanisms of action we characterized Mtb’s transcriptional profile in response to sALT629 and compared the gene expression signature with previous datasets. Briefly, WT Mtb was cultured in medium containing butyrate and treated with 25 μM sALT629 or DMSO for 4 h. In response to sALT629, Mtb induced 354 genes (Log_2_FC>2, *p* < 0.05) and repressed 51 genes (Log_2_FC<−2, *p* < 0.05) (Table S1 and Fig. S1). Overall, the Mtb transcriptional profile produced in response to sALT629 displayed little overlap with the signatures associated with general antitubercular compounds (29) or cell wall synthesis inhibitors (30). Additionally, sALT629 appeared to not perturb bioenergetic pathways (31), induce thiol stress (32), nor disrupt pH homeostasis (29). Among the most upregulated genes following exposure to sALT629 are genes encoding proteins thought to be involved in small molecule detoxification/modification including the monooxygenase *ethA* and the benzoquinone methyltransferase *rv0560c* (33). Of the 51 most downregulated genes, 22 are regulated by DosRST which are typically induced in response to nitric oxide or hypoxia (34). Altogether, the transcriptional response following sALT629 treatment suggests a putative mechanism of action distinct from other previously described antitubercular compounds.

### Mutations in HadC confer sALT629 resistance

**Figure 2:**
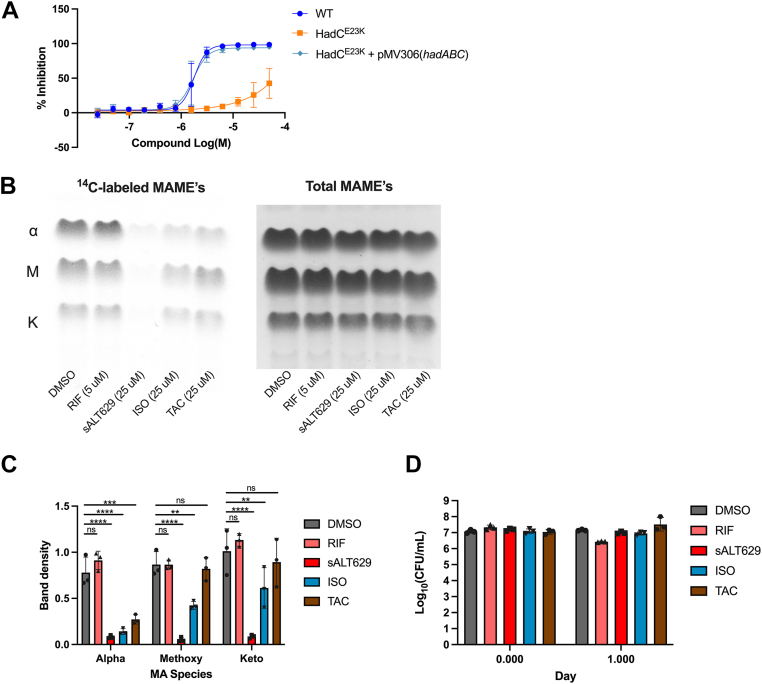
**A HadC^E23K^ mutation confers sALT629 resistance and mycolic acid synthesis is perturbed by sALT629.***A*, sALT629 dose-response inhibition curve with WT, HadC^E23K^, or complement HadC^E23K^ + pMV306(*hadABC*) in 7H12 butyrate media. Data are from two biological replicates with three technical replicates, each (n = 6). *Error bars* represent SD. *B*, TLC images of ^14^C-labeled ΜΑΜΕ’s and total ΜΑΜΕ᾽s from WT Mtb treated with the indicated inhibitors or DMSO. Equal volumes of each sample were loaded. TLC images are representative of three independent replicates. α = alpha mycolic acids, M = methoxy mycolic acids, k = keto mycolic acids. *C*, densitometry quantification for the ratio of ^14^C-labeled ΜΑME᾽s to total ΜΑΜΕ᾽s for each sample (n = 3). *Error bars* represent the average ± SD. Samples were analyzed by two-way ANOVA with Dunnett’s multiple comparisons test (∗∗∗∗*p* < 0.0001; ∗∗∗*p* ≤ 0.001; ∗∗*p* ≤ 0.01; ns = not significant). *D*, bacterial viability was quantified under the conditions used for ^14^C-labeling. Data are from three biological replicates (n = 3) and *error bars* represent SD. Samples were analyzed by two-way ANOVA with Dunnett’s multiple comparisons test but were not significantly different from the DMSO control. MAME, mycolic acid methyl ester.

To investigate sALT629’s mechanism of action, we isolated spontaneous resistance mutants by plating ∼10^6^ WT Mtb on agar plates containing an analog of sALT629 (mCNU349) (Fig. 1). We used mCNU349 to select resistant mutants because we could not obtain resistant mutants on plates containing sALT629. A small number of colonies (∼58) were observed after 2 months of culture. Of these, 15 colonies were isolated and assayed against sALT629 using an Alamar blue-based microplate MIC assay. One clone was resistant to sALT629 up to a concentration of 50 μM. Whole genome sequencing of the resistant clone revealed a mutation located in the *hadC* gene resulting in an amino acid substitution (E23K) in the HadC protein. To confirm that this mutation was responsible for resistance to sALT629, we introduced a WT copy of the *hadABC* operon expressed from its native promoter (pMV306(*hadABC*)) and fully restored susceptibility of this strain to sALT629 (Fig. 2*A*).

HadC is required to generate long-chain keto- and methoxy-mycolic acids (35). Thus, we determined if sALT629 inhibited *de novo* biosynthesis of mycolic acids similar to the HadAB inhibitors thioacetazone (TAC) and ISO (36, 37, 38). Mtb was simultaneously exposed to the inhibitor compounds and metabolically labeled with [^14^C]-acetate before analyzing the mycolic acid methyl ester (MAME) profiles. We found that sALT629 inhibited *de novo* synthesis of α-, keto-, and methoxy-mycolic acid species while TAC and ISO treatments primarily inhibited the synthesis of α-mycolic acids in this assay (Fig. 2, *B* and *C*). The inhibition of ^14^C-acetate incorporation into *de novo* mycolic acid synthesis was not associated with bacterial killing by sALT629 over the 24-h period of the labeling experiment (Fig. 2*D*). Finding that sALT629 perturbs mycolic acid synthesis was surprising given that Mtb’s transcriptional signature in response to sALT629 is not associated with an induction of *fabD*, *acpM*, *kasA*, *kasB*, and *accD6* since these genes are typically induced by Fas2 inhibitors (Fig. S1) (29). The dramatic inhibition of ^14^C-acetate incorporation into *de novo* synthesis of all mycolic acid species by sALT629 suggests that this compound targets a process upstream of the Fas2 complex.

### The HadC^E23K^ mutation does not compensate for loss of HadA

Mutations that enhance the processivity or activity of the HadBC dehydratase complex confer resistance to inhibitors targeting HadAB (39). Thus, we sought to determine if the HadC^E23K^ mutation can suppress genetic defects engineered into HadA. To do this, we integrated a second copy of the WT *hadABC* operon at the L5 site of the Mtb genome (WT*-hadABC*). Separately, we introduced a mutated version of the *hadABC* operon at the L5 site to express WT HadB, the HadC^E23K^ variant and a copy of the *hadA* gene that carries a frameshift mutation rendering HadA nonfunctional (MUT*-hadA*^*fs*^*BC*^*E23K*^). Using these two strains we next attempted to delete the native *hadABC* operon in the Mtb genome by allelic exchange. While we could readily delete the native *hadABC* operon in the WT*-hadABC* strain we were unable to detect any allelic exchange at the native *hadABC* operon in the MUT*-hadA*^*fs*^*BC*^*E23K*^ strain (Table S2). Only six clones arose when we attempted to delete native *hadABC* operon in the MUT*-hadA*^*fs*^*BC*^*E23K*^ strain. Using Sanger sequencing we confirmed that all six of these clones carried WT alleles at the native *hadABC* operon site and these antibiotic-resistant clones arose due to illegitimate recombination elsewhere in the genome. These results indicated that the HadC^E23K^ variant did not stabilize the HadBC complex in a manner that could compensate for loss of HadA activity. Thus, we conclude that sALT629 is likely not targeting the HadAB complex. This interpretation was reinforced by confirming inhibitory activity of sALT629 in strains that are resistant to ISO due to mutations in *hadA* and *hadC* (Table S3).

## sALT629 disrupts the global lipidome in Mtb

**Figure 3:**
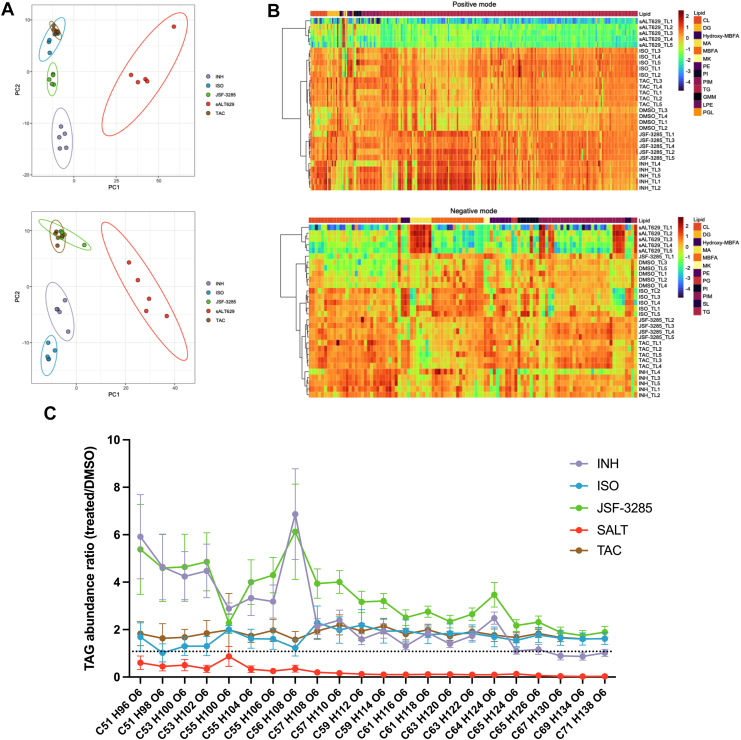
**sALT629 treatment depletes TAG levels and is differentiated from known Fas2 inhibitors.***A*, PCA of statistically significant lipid features detected in positive (*top*) and negative (*bottom*) mode that differed in abundance between sALT629 treatment and all canonical Fas2 inhibitors used. PCA plots are based on features filtered by |logFC| ≥1 and *P*_adj_ < 0.05. *B*, hierarchical clustered heat maps of statistically significant lipid features detected in positive (*top*) and negative (*bottom*) mode that differed in abundance between sALT629 treatment and all canonical Fas2 inhibitors used. Lipid abundances in the heat maps were Log_2_-transformed and centered and scaled such that the amounts (−4 to 2) are in a Gaussian distribution for each feature. CL, cardiolipin; DG, diacylglycerol; hydxroxy-MBFA, hydroxy-methyl-branched fatty acids; mycolic acid, mycolic acid; MBFA, methyl-branched fatty acids; MK, menaquinone; PE, phosphatidylethanolamine; PI, phosphatidylinositol; PG, phosphatidylglycerol; PIM, phophosphatidylinositol mannoside; TG, triacylglycerol; GMM, glucose monomycolate; LPE, lysophosphatidylethanolamine; PGL, phenolic glycolipid; SL, sulfolipid. *C*, relative abundance of representative TAG species detected in positive ion mode and quantified with Skyline. Data are from four or more independent replicates; error bars represent the average ± SD.

The dramatic inhibition of *de novo* mycolic acid synthesis by sALT629 suggested that sALT629 inhibits a process preceding Fas2 in Mtb. Thus, we characterized the global lipidome in Mtb following sALT629 treatment. To differentiate sALT629 from known Fas2 inhibitors we also profiled Mtb’s response to various different Fas2 inhibitors; INH, ISO, TAC, and JSF-3285 which inhibits KasA, the Fas2 β-ketoacyl synthase (40). In these studies, WT Mtb was exposed to the inhibitors for 24 h before isolating and analyzing total bacterial lipids by LC-MS. A total of 3836 and 3718 lipid features (defined by rt and *m/z*) were detected by positive and negative mode ionization, respectively (Table S4). Principle component analyses of the normalized abundances for each feature demonstrated a clustering of the Fas2 inhibitor treatment groups while sALT629 formed its own cluster (Fig. S2). Features that showed a significant difference (adjusted *p* < 0.05) in comparisons between the DMSO control and any treatment group were subjected to a univariate analysis of sALT629 against all other treatment groups. This resulted in 669 positive mode and 425 negative mode features that significantly differed in abundance between sALT629 treatment and all canonical Fas2 inhibitors tested (Table S5) and (Fig. 3*A*). The lipid features in this data set were interrogated against Mtb LipidDB (41) to provide lipid identifications (Table S6) and visualized (Fig. 3*B*).

Interrogation of the annotated data revealed that while the abundances of many lipid species were altered upon sALT629 treatment there was a dramatic depletion of TAG species to levels below the DMSO treatment (Fig. 3, *B* and *C*). This led us to hypothesize that sALT629 inhibits *de novo* fatty acid synthesis and Mtb mobilizes acyl chains from TAG to support phospholipid and mycolic acid biosynthesis in the presence of sALT629. Additionally, treatment with Fas2 inhibitors resulted in the accumulation of most TAG species (Fig. 3*C*). This suggests that TAG serves as a deposit for excess mycolic acid α-chains and/or acyl chains that are unable to undergo elongation to full length when Fas2 is perturbed.

### sALT629 inhibits *de novo* fatty acid synthesis in Mtb

**Figure 4:**
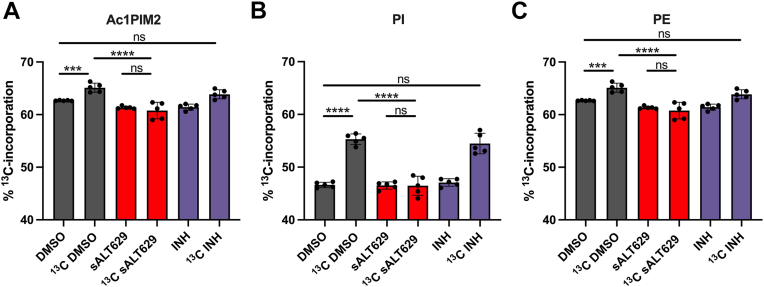
**Incorporation of ^13^C-acetate into phospholipid biosynthesis is inhibited by sALT629.** Total lipids were analyzed by LC-MS in negative ion mode to assess ^13^C-acetate incorporation in the presence of DMSO, sALT629 (25 μM), or INH (25 μM). *A*, Ac1PIM2 (R1CO2H + R2CO2H + R3CO2H+ = C51:0, R4 = H) [M-H]-. *B*, PI (R1CO2H + R2CO2H = C35:0) [M-H]-. *C*, PE (R1CO2H + R2CO2H = C35:0) [M-H]-.

We next examined the incorporation of metabolically labeled acetate into Mtb phospholipid species in the presence of sALT629. Briefly, cultures of WT Mtb were supplied ^13^C-acetate and DMSO, sALT629 or INH for 24 h, then the lipid products were isolated by solvent extraction. We used LC-MS to monitor the incorporation of ^13^C-acetate into *de novo* synthesis of phospholipids in WT Mtb treated with DMSO, sALT629, or INH. We could readily detect the incorporation of ^13^C-acetate into acylated phosphatidylinositol mannoside, phosphatidylinositol (PI), and phosphatidylethanolamine (PE) following DMSO and INH treatment as evident from the altered isotope distribution patterns demonstrating additional ^13^C-labeled atoms beyond the amount of naturally occurring ^13^C atoms in the lipid (Fig. 4, *A*–*C*) and (Fig. S3). Importantly, the incorporation of ^13^C-aceteate into Ac1Pim2, PI, and PE was blocked with sALT629 treatment.

### The HadC^E23K^ mutation results in loss of function and alters fatty acid distribution in Mtb

**Figure 5:**
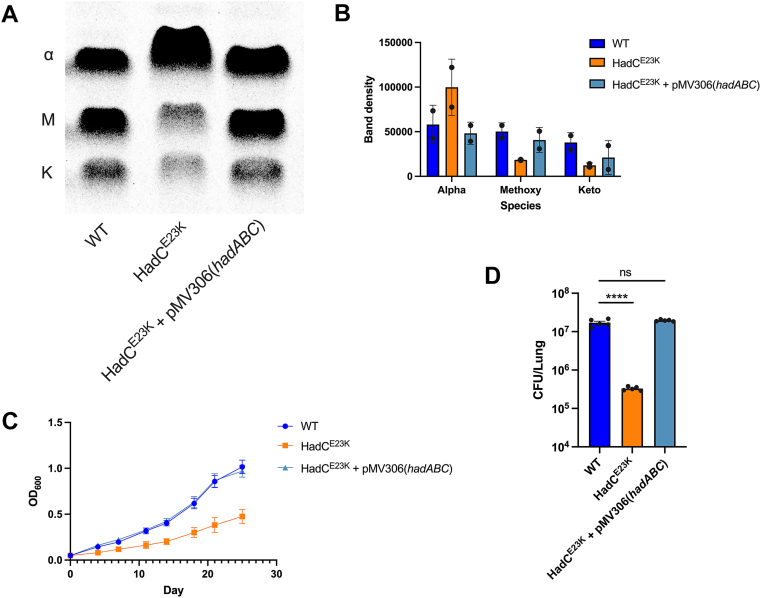
**The HadC^E23K^ protein is inactive and confers a fitness defect *in vitro* and *in vivo*.***A*, radio-TLC image depicting ^14^C-labeled MAME’s from untreated bacteria. Equal counts for each sample were loaded (25,000 CPM/sample). α = alpha mycolic acids, M = methoxy mycolic acids, k = keto mycolic acids. *B*, densitometry-based quantification ^14^C incorporation into the different MAME species. The TLC image is representative of two independent replicates (n = 2). *Error bars* represent the average ± SD. *C*, bacterial growth in 7H12 butyrate media, data from three independent replicates. *D*, bacterial burdens from the lungs of BALB/c mice 7 weeks after aerosol challenge with ∼200 CFU’s. Five mice were used for each group and *error bars* represent the average ± SD. Data were analyzed with one-way ANOVA and Dunnett’s multiple comparisons test (∗∗∗∗*p* < 0.0001). MAME, mycolic acid methyl ester.

It is unusual that mutations in HadC, a nonessential dehydratase subunit of the Fas2 pathway, confers resistance to sALT629. HadC contains the amino acid residues essential for interactions with acyl-AcpM and to mediate enzyme complex formation with the catalytic subunit HadB (42). The HadBC complex is thought to be involved in late cycles of Fas2-mediated synthesis to generate long acyl chains that are modified to keto- or methoxy-mycolic acids (35, 43). Thus, we evaluated *de novo* mycolic acid synthesis in the HadC^E23K^ mutant with ^14^C-acetate labeling to determine if this resulted in a HadC loss of function. The HadC^E23K^ mutant exhibited a striking decrease in keto- and methoxy-mycolic acid species with a dramatic increase in the levels of α-mycolic acid (Fig. 5, *A* and *B*). This finding is consistent with previous studies which demonstrated that deletion mutants lacking HadC produce decreased amounts of keto- and methoxy-mycolic acids with a compensatory increase in α-mycolic acids (35, 39). Previous studies also reported that HadC deletion mutants display fitness defects during infection, antibiotic exposure, and SDS-mediated cell wall stress (35). To further confirm that the HadC^E23K^ mutation results in a loss of function we characterized growth of the mutant in broth medium and observed a pronounced growth defect (Fig. 5*C*). We also determined that the HadC^E23K^ mutant is attenuated in mice resulting in a ∼2 log_10_ reduction in lung bacterial burdens compared to the WT and complement strain 7 weeks after aerosol challenge (Fig. 5*D*). Furthermore, we have found that the HadC^E23K^ mutation is associated with an elevated sensitivity to bedaquiline and rifampicin (Fig. S4). Together, these data indicate that HadC loss of function mutations confers resistance to sALT629 and that this class of mutation would likely not evolve in a clinical setting especially in the presence of treatments including bedaquiline or rifampicin.

**Figure 6:**
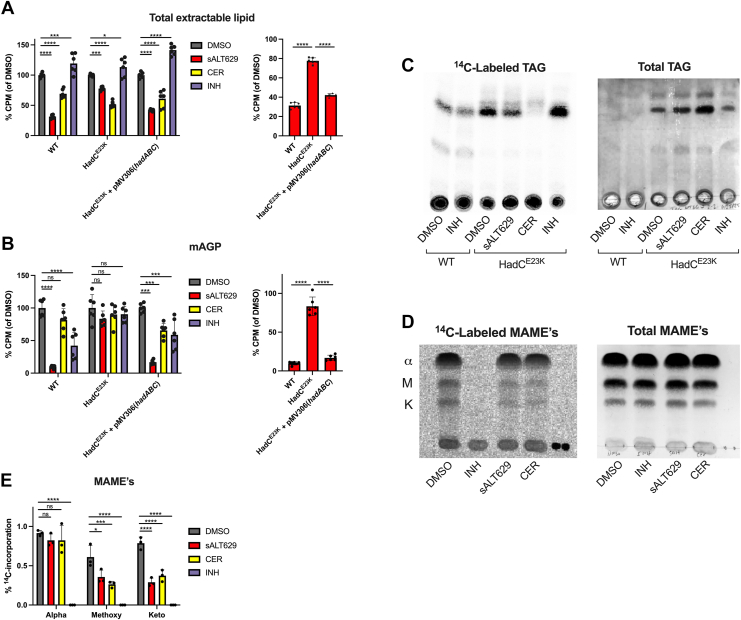
**The HadC^E23K^ mutation restores lipid synthesis and accumulates TAG.***A*, ^14^C-acetate incorporation into total extractable lipids (all treatments, *left*; only sALT629, *right*). *B*, ^14^C-acetate incorporation into the delipidated cell or mAGP fraction (all treatments, *left*; only sALT629, *right*). Data are from three independent biological replicates with two technical replicates each (n = 6) and data are normalized to the untreated control. *Error bars* represent the average ± SD. Samples were analyzed by two-way ANOVA (*left*) or one-way ANOVA (*right*) with Dunnett’s multiple comparisons test (∗∗∗∗*p* < 0.0001; ∗∗∗*p* ≤ 0.001; ∗*p* ≤ 0.05). *C*, TLC images of ^14^C-labeled and total TAG species extracted from WT and the HadC^E23K^ treated with inhibitors or DMSO. *D*, TLC images of ^14^C-labeled ΜΑΜΕ’s and total ΜΑΜΕ᾽s from WT Mtb treated with the indicated inhibitors or DMSO. Equal volumes of each sample were loaded. TLC images are representative of three independent replicates. α = alpha mycolic acids, M = methoxy mycolic acids, k = keto mycolic acids. *E*, densitometry quantification for the ratio of ^14^C-labeled ΜΑME᾽s to total ΜΑΜΕ᾽s for each sample (n = 3). Error bars represent the average ± SD. Samples were analyzed by two-way ANOVA with Dunnett’s multiple comparisons test (∗∗∗∗*p* < 0.0001; ∗∗∗*p* ≤ 0.001; ∗∗*p* ≤ 0.01; ns = not significant). TLC images are representative from three independent replicates. Strains were treated with sALT629 25 μM, CER 45 μM, or INH 25 μM. MAME, mycolic acid methyl ester.

Our lipidomics and metabolic labeling data indicate that sALT629 inhibits the *de novo* synthesis of acyl chains in Mtb and this drives TAG consumption. Thus, we predicted that the HadC^E23K^ mutant could redirect acyl chains originally destined for long chain keto- and methoxy-mycolic acids biosynthesis into other arms of Mtb lipid metabolism such as TAG biosynthesis. We further posit that these “aborted” or redirected Fas2 acyl chains are scavenged by Mtb to compensate for shortages in acyl chain availability when *de novo* lipid synthesis is blocked in the HadC^E23K^ mutant. To test this concept, we quantified the distribution of ^14^C-acetate label into total extractable lipids or mycolic acid-arabinogalactan-peptidoglycan complex (mAGP) derived from delipidated cells in the WT, HadC^E23K^ mutant, and complement. In this assay, the distribution of the radiolabeled tracer reflects the predominate biosynthetic products of Fas1 (total lipids) or Fas2 (mAGP) (44). Briefly, WT Mtb was simultaneously treated with ^14^C-acetate and sALT629, cerulenin, or INH for 24 h before the lipid products were isolated by solvent extraction. We quantified the relative level of ^14^C-incorporation by scintillation counting. In the presence of sALT629 the incorporation of ^14^C-acetate into total lipids was significantly reduced in WT Mtb and this reduction was almost completely reversed in the HadC^E23K^ mutant (Fig. 6*A*). Cerulenin is known to inhibit the mycobacterial Fas1 and Fas2 systems (45) and in this assay cerulenin inhibits the incorporation of ^14^C-acetate into total lipids in WT. Importantly, the cerulenin-dependent inhibition of ^14^C-acetate incorporation into total lipids is not reversed in the HadC^E23K^ mutant suggesting that cerulenin and sALT629 possess different mechanisms of action (Fig. 6*A*). As expected, INH treatment selectively inhibited incorporation of ^14^C-acetate into the mAGP but not total lipids in WT bacteria (Fig. 6*B*). Analysis of ^14^C-label incorporation into mAGP fractions from the HadC^E23K^ confirmed a redistribution of the radiolabel into mAGP in the mutant, in the presence of sALT629 (Fig. 6*B*). We reasoned that resistance to sALT629 is associated with increased TAG levels in the HadC^E23K^ mutant. To test this, we assessed the incorporation of ^14^C-acetate into TAG’s in the HadC^E23K^ mutant and observed an accumulation of absolute levels of TAG and restoration of ^14^C-incorporation into *de novo* synthesized TAG in the presence of sALT629 in the mutant compared to the WT (Fig. 6*C*). The abundance of free fatty acids and TAG in the mutant suggests that TAG-derived acyl chains are directly used to maintain free fatty acid and phospholipid pools when *de novo* fatty acid synthesis is blocked by sALT629 in the HadC^E23K^ mutant. Exposing WT Mtb to sALT629 dramatically reduces the incorporation of ^14^C-acetate into *de novo* synthesized mycolic acid species (Fig. 1*B*). Our model proposes that resistance to sALT629 involves redirecting acyl chains from TAG into Fas1 and Fas2 pathways. If this is correct we predict that ^14^C-acetate will accumulate in *de novo* syntheized mycolic acids in the HadC^E23K^ mutant in the presence of sALT629. Thus, we quantified the amount of ^14^C-labeled acetate incorporated into the mycolic acids in the HadC^E23K^ mutant. We found that even in the presence of sALT629, the HadC^E23K^ mutant incorporates ^14^C- acetate into α-mycolic acids acids to levels similar to the DMSO control (Fig. 6, *D* and *E*). The HadC^E23K^ mutant still incorporated less ^14^C-acetate into methoxy- and keto-mycolates in the presence of sALT629. The accumulation of ^14^C-labeled α-mycolic acids in the HadC^E23K^ mutant provides more evidence for an interconnection between TAG pools and lipid synthesis pathways in Mtb. The lack of significant ^14^C-acetate incorporation in the oxygenated mycolic acid species in the HadC^E23K^ mutant was expected given the biosynthesis defect in this mutant.

### Fatty acids, pantothenate, and biotin fail to abrogate sALT629-mediated inhibition.

We next evaluated if sALT629-mediated inhibition of *de novo* fatty acid synthesis could be rescued by scavenging exogenous C_18_-C_24_ acyl chains. We found that supplementing Mtb cultures with high concentrations of oleate (C_18:1_) or behenic acid (C_22_) did not rescue sALT629 inhibition (Fig. S5*A*). To rule out if sALT629 targets pantothenate synthesis, a pathway required for CoA biosynthesis, we confirmed that supplementation with pantothenate did not rescue Mtb inhibition by sALT629 (Fig. S5*B*). Similarly, supplementation with biotin, a cofactor required for the conversion of acetyl-CoA to malonyl-CoA by acetyl-CoA carboxylases, failed to rescue Mtb inhibition by sALT629 (Fig. S5*C*). The data presented thus far suggest that sALT629 targets Fas1 activity or an ancillary reaction like Fas1 activation (P-pant addition).

## Discussion

TB drug discovery efforts are focused on identifying antitubercular compounds with new mechanisms of action without preexisting clinical resistance. We initially identified sALT629 in a screen for compounds that inhibit Mtb growth in macrophages and confirmed that sALT629 is active against Mtb in various conditions including media containing host-relevant carbon sources. Transcriptional profiling studies with sALT629 indicate that this compound has a mechanism of action unlike other Mtb inhibitors or drugs with known targets. By leveraging lipidomic analysis and monitoring the incorporation of metabolic tracers into bacterial lipid products we establish that sALT629 inhibits *de novo* fatty acid synthesis in Mtb. One notable feature in our lipidomic analysis was the drastic depletion of TAG species in response to sALT629. This observation is very similar to what has been reported in *Mycobacterium smegmatis* upon genetic knockdown of Fas1 leading to a dramatic reduction in cellular TAG pools (46). Altogether, this suggests that when *de novo* fatty acid synthesis is inhibited, TAG acyl chains are scavenged to maintain synthesis of critical lipids such as phospholipids and/or mycolic acids. It is important to note that our experimental approach was unable to account for a potential sALT629-mediated inhibition of TAG synthesis in Mtb. While the reduction in the absolute levels of TAG reflects some level of TAG consumption, it is also likely that *de novo* TAG synthesis is inhibited in the presence of sALT629.

We discovered that a loss of function HadC^E23K^ mutant accumulates TAG and this likely contributes to sALT629 resistance. We hypothesize that this mutant diverts acyl chains fated for Fas2-mediated elongation into TAG. The mechanism by which this occurs remains to be elucidated but a link between perturbations to Fas2 and TAG levels exists. It is well documented that Fas2 inhibitors such as INH lead to accumulation of ∼C_22_-C_26_ acyl chains in mycobacteria (47). Subinhibitory concentrations of INH also induce the formation of a TAG-related lipid termed meromycolyl-diacylglycerol which contains two standard acyl chains, and a meromycolic acid precursor in *Mycobacterium kansasii*. Moreover, subinhibitory INH treatment induces the formation of visible lipid droplets in *M. kansasii*, suggesting that meromycolyl-diacylglycerol accumulates in bacterial lipid droplets upon Fas2 perturbations (48). Blocking of Fas2 with various inhibitors results in the accumulation of TAG species suggesting that Fas2 perturbations result in the formation of TAG species containing acyl chains originally destined to Fas2-mediated elongation. Thus, TAG likely serves as an acyl chain depository when lipid synthesis pathways in Mtb are perturbed.

The HadC^E23K^ mutant identified in these studies represents an indirect mechanism of resistance to sALT692. Mutations of HadC’s amino acids 85, 123, 151, and 157 are associated with ISO and TAC resistance (36, 38, 39). However, the specific HadC^E23K^ mutation that promotes resistance to sALT629 is not associated with ISO or TAC resistance and our data indicate that the HadC^E23K^ protein is inactive and that this mutant has a dramatically altered mycolic acid profile. The specific HadC variants HadC^V85I^ and HadC^K157R^ form a functional complex with HadB and can compensate for genetic inactivation of HadA (39). We could not detect any HadA compensation associated with the HadC^E23K^ mutation. The mechanism by which HadC^E23K^ confers resistance to sALT629 is distinct from previously described ISO and TAC resistance mutations and is perhaps related to the accumulation of TAG observed in this strain. Importantly, the mutant’s observed fitness defect and increased sensitivity to rifampicin (RIF) and bedaquiline suggest that this mechanism of resistance is unlikely to occur in a clinical setting.

It is well established that TAG levels in Mtb are influenced by environmental conditions such as hypoxia or nitric oxide exposure, and this is mediated by the DosRST system (49, 50, 51). DosR directly regulates *tgs1*, which encodes a TAG synthase (TGS) involved in the last step of TAG biosynthesis which is required for TAG accumulation during hypoxia (34, 50, 52). Our transcriptional profiling revealed that most genes in the DosR regulon including *tgs1* were strongly downregulated upon sALT629 treatment in Mtb (Fig. S1). It is unlikely that sALT629 inhibits TAG biosynthesis because within 24 h, treatment with sALT629 reduces TAG abundance to levels below that is observed in untreated bacteria. This is consistent with sALT629-mediated TAG turnover or consumption rather than inhibition of TAG biosynthesis. Additionally, the high levels of enzyme redundancy (15 total TGS enzymes encoded by the Mtb genome) suggest that it is unlikely for sALT629 to inhibit TAG synthesis (50). Thus, we interpret the reduction in DosR regulon expression as a compensatory mechanism to halt TAG synthesis in Mtb in response to sALT629 in order to salvage pre-formed TAG acyl chains for phospholipid and/or mycolic acid synthesis.

It is plausible that Mtb may circumvent sensitivity to sALT629 by importing host derived ∼ C_20_-C_26_ acyl chains. We found that supplementation with oleate (C18:1) or behenic acid (C22:0) did not affect the MIC of sALT629. Moreover, it is unlikely that Mtb would have access to saturate ∼ C_22_-C_26_ fatty acids within host cells or tissues; while such fatty acids are present in mammals, they are less abundant than C_11_-C_20_ fatty acids (53, 54). Additionally, sALT629 has anti-Mtb activity inside the macrophage suggesting that the intracellular host environment may not supply either the correct species or sufficient amounts of acyl chains needed to rescue lipid synthesis in the presence of sALT629.

While our data demonstrate that sALT629 inhibits *de novo* fatty acid synthesis, the precise target remains to be elucidated. Fatty acid biosynthesis is contingent on several ancillary reactions. First, CoA is required for producing malonyl-CoA. Biosynthesis of CoA is an essential process in Mtb and involves the intermediate pantothenate (26). We found that supplementation with 1 mM pantothenate did not rescue sALT629 inhibition. If CoA biosynthesis is inhibited by sALT629, it likely occurs downstream of the pantothenate generating step. Second, malonyl-CoA is generated *via* the biotin-dependent carboxylation of acetyl-CoA *via* ACC activity (25, 55). Additionally, biotin biosynthesis in Mtb is dependent on the synthesis of a Fas1-derived C_7_ intermediate. We found that supplementation with 100 uM biotin did not rescue sALT629 inhibition. Thus, biotin biosynthesis is unlikely targeted by the compound. It is worth noting that malonyl-CoA is converted to malonyl-ACP which serves as the extender for Fas2 elongation cycles and Fas2 appears to be partly functional in the presence sALT629 treatment in the HadC^E23K^ mutant (Fig. 5*B*), thus ACC itself seems unlikely to be the target of sALT629. Finally, fatty acid biosynthesis requires the shuttling of nascent acyl chains by an ACP domain in Fas1 or on AcpM for Fas2 (56, 57, 58). ACP is activated by the addition of 4′-phosphopantetheine by PPTases (AcpS for Fas1 and PptT for AcpM) (24, 59, 60). It is possible that sALT629 inhibits the addition of pantetheine to Fas1 by AcpS; this requires further investigation. Finally, Fas1 itself is a multi-domain enzyme (61), providing many options for potential targets of sALT629. Identifying the precise enzymatic target of sALT629 will likely reveal a drug target that has not yet been exploited in Mtb.

While the target of sALT629 is promising, the compound itself requires further optimization. The inhibitory activity of sALT629 tapers after ∼7 days of compound exposure (submitted manuscript). Thus, additional resistance mechanisms may exist or sALT629 could be modified by one or more enzymes induced in response to sALT629 treatment and this could deactivate the compound. Lead optimization efforts may improve killing kinetics and potentially or reduce any modification/inactivation. Overall, this work has characterized Mtb’s physiological response associated with inhibition of *de novo* fatty acid synthesis. These studies will enable future efforts to target this aspect Mtb biology and to identify more compounds with modes of action similar to sALT629.

## Experimental procedures

### Bacterial strains and media

*M. tuberculosis* Erdman was used for all experiments, unless otherwise noted. *M. tuberculosis* H37Rv mc^2^6206 was used where indicated. Bacteria were routinely grown in Middlebrook 7H9 media supplemented with 0.2% glycerol and 10% OADC supplement (BD), hereafter referred to as 7H9 OADC. 7H9 ADC media contained 7H9 supplemented with 0.2% glycerol and 10% ADC supplement. 7H12 medium is 7H9 liquid media supplemented with 0.1% casitone and 100 mM MES free acid monohydrate, pH 6.6. Where indicated, bacteria were grown in 7H12 + 0.1% sodium acetate (7H12 acetate), 7H12 + 0.1% sodium butyrate (7H12 butyrate) or 7H12 + 100 μM cholesterol (7H12 cholesterol). Cholesterol that was used to supplement liquid media was solubilized in tyloxapol:ethanol (1:1, v/v). All liquid media contained tyloxapol (Thermo Fisher Scientific) at a final concentration of 0.05%.

### Inhibition assays

Assays were performed using black, clear-bottom 96-well plates. All compounds were titrated using 2-fold dilutions, and RIF (10 μM) was used as the positive control and DMSO as the negative control. The plates were inoculated at an OD_600_ of 0.01 and incubated at 37 °C in a 6% CO_2_ environment for 9 days. To quantify bacterial inhibition, 40 μl of sterile Alamar blue (50% in water) was added to each well and the plates were incubated for an additional 24 h. To quantify Alamar blue reduction, fluorescence (492 nm excitation: 595 nm emission) was read using a PerkinElmer Envision plate reader. The absorbance data were normalized to the RIF control (100% inhibition) and analyzed with GraphPad Prism to generate % inhibition curves and EC_50_ values.

### Bacterial killing assays

To assess bactericidal activity of sALT629, WT Mtb Erdman was inoculated at an OD_600_ of 0.12 in 5 ml of 7H12 butyrate containing compounds at the indicated concentration or DMSO in vented T-25 flasks. The flasks were incubated standing at 37 °C in a 6% CO_2_ environment. After incubating for 5 days culture aliquots were collected, diluted, and plated onto 7H10 OADC plates for colony forming units (CFU) enumeration.

### Transcriptional profiling

Bacteria were grown in 7H9 OADC to mid-log phase and diluted to an OD_600_ of 0.35 in vented T-25 flasks. Inhibitors were added to the concentration of 25 μM in triplicate and cultures were incubated in standing vented T-25 flasks at 37 °C in a 6% CO_2_ environment. After a 4 h incubation the bacteria were isolated by centrifugation and resuspended in Trizol LS (Ambion). Samples were lysed by bead beating and the RNA was isolated by chloroform extraction, isopropanol precipitation, and resuspended in nuclease-free water. Sequencing was performed by SeqCenter after samples were treated with Invitrogen DNAse (RNAse free). Ribosomal RNA was depleted using custom Mtb specific oligonucleotides and library preparation was performed using Illumina’s Stranded Total RNA Prep Ligation and 10 bp unique dual indices. Sequencing was done on a NovaSeq X Plus device, producing paired end 150 bp reads. The reads were de-multiplexed and adapter sequences were trimmed with bcl-convert (v4.1.5). Paired-end sequence mapping to the H37Rv genome (accession number GCA_000195955.2) was performed with STAR-2.7.10 b. HTSeq v2.02 with H37Rv annotations was used to obtain raw counts. R was used for read counts normalization and differential expression analysis *via* DESeq2 v1.44.0 and APEGLM v1.26.1 for log-fold change estimation. Genes with <10 raw counts were excluded from further analysis and the resulting genes were filtered for FDR<0.05 and |FC|>1. The raw RNAseq data set generated in this study has been deposited to the NCBI Gene Expression Omnibus (accession number: GSE298382).

### Screening and identification of spontaneous resistance mutants

Approximately 10^6^ CFU of a WT Mtb culture was plated on 7H10 agar containing 0.1% sodium butyrate and 10 μM or 25 μM of mCNU349 (a sALT629 analog) or sALT629. Plates were incubated at 37 °C for approximately 2 months to allow slow-growing colonies to appear. No colonies grew on the sALT629 plates but fifteen colonies from the mCNU349 plates were picked and screened with an Alamar blue-based inhibition assay as described.

To obtain a WT reference genome sequence, a representative WT Mtb culture was streaked for individual colonies on 7H10 OADC agar. Genomic DNA was isolated from one WT colony and submitted for whole-genome sequencing. This WT DNA sequence was assembled using the reference genome (NCBI accession GCA_000350205.1). Genomic DNA was isolated from one spontaneous resistance clone for Illumina whole-genome sequencing. Genomic DNA sequencing was performed at SeqCenter. Sample libraries were prepared using the Illumina DNA Prep kit and IDT 10 bp unique dual indices indices, and sequenced on an Illumina NextSeq 2000 device, producing 2 × 151 bp reads. De-multiplexing, quality control and adapter trimming were performed with bcl-convert (v3.9.3). The resulting 2 × 151 bp paired-end read data was used as the input for variant calling against the WT control genomic DNA sequence.

### Metabolic labeling

To assess *de novo* synthesis of fatty acid and mycolic acid, the bacterial strains were grown in 7H12 butyrate at 37 °C, 6% CO_2_ in standing, vented T-25 flasks. Upon reaching an OD_600_ 0.25 to 0.4 the cultures were pulsed with 10 μCi of [1,2-^14^C] acetate or 500 μM of [1,2-^13^C] acetate in the presence of the indicated compounds for 24 h. The isotopically labeled bacterial cultures were pelleted and the cells were extracted with chloroform:methanol (2:1, v/v) as described.

#### Total lipid extraction and MAME generation

Total extractable lipids were isolated from Mtb by extracting the bacterial cell with chloroform:methanol (2:1, v/v) three times as described (44, 62). The pooled solvent extractable layers were dried to completion under a N_2_ bath. The remaining delipidated bacterial pellets (mAGP) were also dried to completion under a N_2_ bath and used to generate MAMEs. For this, the dry delipidated mAGP pellets were suspended in 15% tert-butyl-ammonium-hydride and saponified at 70 °C overnight (63). To generate MAMEs, dichloromethane and iodomethane were added to the saponified samples and rotated for 1 h at room temperature. The dichloromethane solvent phase was collected, transferred to a new tube and dried to completion under a N_2_ bath. To separate out the tert-butyl-ammonium-hydride salts, the samples were resuspended in diethyl ether, vortexed, centrifuged and the diethyl ether layer was transferred to a new tube and dried under a nitrogen bath.

To evaluate bacterial killing in the *de novo* mycolic acid synthesis experiments, Mtb cultures were grown in parallel conditions in 30 ml of 7H12 butyrate to OD_600_ 0.3 to 0.4 and split into T-25 flasks containing inhibitors or DMSO without ^14^C-radiolabel. The cultures were incubated in standing, vented T-25 flasks at 37 °C in a 6% CO_2_ environment. After 24 h bacterial aliquots were collected, diluted and plated onto 7H10 OADC. Final CFU counts were obtained after 4 weeks of incubation at 37 °C.

### Analysis of the total extracted lipids and MAMEs

To visualize the ^14^C-labeled TAG species, the total extracted lipids were resolved on an aluminum-backed silica gel TLC plate using a mobile phase solvent mixture of toluene:acetone (99:1, v/v). The ^14^C labeled MAME’s were resolved on an aluminum-backed silica gel TLC plate using a mobile phase solvent mixture of petroleum ether:diethyl ether (95:5, v/v) eight times. Visualization of ^14^C-labeled species was achieved by exposing TLCs to a storage phosphor screen and imaging with a PhosphorImager. Total triacylglycerol and mycolic acids resolved on the TLCs were visualized by spraying and charring using 5% phosphomolybdic acid in ethanol (52).

#### Strain construction to evaluate HadC^E23K^ compensation for a HadA deficiency

The WT-*hadABC* strain was generated by cloning the entire *hadABC* operon including the native promoter contained in the 555 bp upstream of *hadA* into the integrating vector pMV306(*hadABC*). The MUT-*hadA*^*fs*^*BC*^*E23K*^ strain was constructed by introducing the mutations into the pMV306(*hadABC*) plasmid using site-directed mutagenesis. Both plasmids were transformed into WT Mtb Erdman. The resulting WT-*hadABC* and MUT-*hadA*^*fs*^*BC*^*E23K*^ strains were grown to mid-log phase, matched to similar OD_600_ values, and made electrocompetent. The plasmid-based allelic exchange substrate used to delete Mtb’s endogenous *hadABC* operon was generated by cloning a hygromycin resistance cassette flanked by ∼1 kb regions upstream of *hadA* and downstream of *hadC.* The allelic exchange plasmid was crosslinked and transformed into electrocompetent Mtb WT-*hadABC* and MUT-*hadA*^*fs*^*BC*^*E23K*^ strains as described (64). To confirm mutants, the native *hadABC* locus and flanking regions were amplified (730,880 bp to 734,402 bp) and sequenced to confirm crossover in the WT strain and lack thereof in the MUT strain.

##### EC_50_ determination with ISO resistant Mtb strains

The susceptibility of *M. tuberculosis* H37Rv mc^2^6206 (Δ*panCD*Δ*leuCD*) to sALT629 was assessed using a resazurin microtiter assay (65) in 96-well plates. Cultures were incubated at 37 °C for 7 days in Middlebrook 7H9 medium supplemented with 0.05% tyloxapol, 10% OADC, 0.2% casamino acids, 48 μg/ml pantothenate, and 50 μg/ml L-leucine for 7 days. EC_50_ values were estimated by visually assessing both the colorimetric change of resazurin (blue to pink) and the extent of bacterial pellet formation.

#### Liquid chromatography-mass spectrometry

Lipid preparations (100 ug) were suspended in 100 μl of chloroform-methanol (1:1, v/v), transferred to LC-MS vials and placed in an autosampler at 8 °C. LC-MS and LC-MS/MS analyses were performed with an Agilent 1290 HPLC and 6546 quadrupole time of flight (Q-TOF) mass spectrometer system (Agilent Technologies) (66). Specifically, 2 μl of the lipid sample were applied to a XBridge C_18_ column (2.1 × 150 mm, 2.5 μm) (Waters Corp.; Milford, MA) *via* autosampler injection. LC separation of the lipids or mycolic acids was achieved by applying a gradient of solvent A (5 mM ammonium acetate in methanol) and solvent B (5 mM ammonium acetate in n-propanol:hexane (80:20, v/v)) at a flow rate of 0.30 ml/min and a column temperature of 45 °C. The LC gradient was 100% solvent A for 3.5 min, followed by a 22.0 min linear gradient to 50% solvent B, and a 10 min linear gradient to 98% solvent B. Solvent B at 98% was maintained for 2 min, followed by a liner gradient to 100% solvent A over 0.5 min, and re-equilibration in 100% solvent A for 6.5 min.

The eluent from LC systems was directly introduced into the Agilent 6546 Q-TOF mass spectrometer equipped with electrospray ionization source. The Q-TOF instrument was operated with the following parameters; capillary voltage, 3500 V; fragmentation voltage, 135 V; drying gas temperature, 310 °C, gas flow, 8 L/min, nebulizer pressure, 40 psig, sheath gas temperature, 270 °C, nozzle voltage, 1000 V and sheath gas flow, 10 L/min. Data were acquired in positive and negative ion mode in separate LC-MS experiments at a MS scan range of 250 - 3000 m/z and MS scan rate of 1.25 spectra/s. Data acquisition and instrument control were conducted by the Agilent MassHunter software. MS/MS analyses were performed with the same instrument parameters except the MS scan rate was 5 spectra/s. The MS/MS scan range and rate were 50 - 3000 m/z and 5 spectra/s, respectively. MS/MS fragmentation was achieved with collision energy of 20 or 35. The Q-TOF mass spectrometer was tuned using tuning solution supplied by Agilent Technologies before sample analyses to ensure optimum mass accuracy (typically <2 ppm error) and resolution.

### Lipidomics data analysis

For untargeted lipidomics data analysis, the Agilent LC-MS data files (.d) were transformed to.mzML open data format using Proteowizard software (67). The mzML data files were applied to the open source MZmine software package for feature extraction (68). The parameters of lipidomics feature extraction are shown in Tables S5 and S6. MZmine feature extraction was performed using all treatment groups (n = 6) and all replicates (n = 5) the output was exported as a.csv and each feature was defined by its *m/z* and retention time (rt) values. One replicate from the DMSO treatment group was removed from the subsequent analyses of the positive mode LC-MS data as it had > 70% missing values. The abundance of each feature was normalized by median fold-change (69). Any feature that was not present in > 2 replicates of any one treatment group was removed. For the remaining features any missing values (peak area abundance) were corrected by a 2-step imputation. 1) Features that were missing from >2 replicates within a group were considered “absent” for that group. Imputation of the “absent” features used one-half minimum imputation. 2) For the remaining missing values kNN imputation was applied. Univariant analyses were performed with the limma package (70); *p*-values were adjusted by the Benjamini-Hochberg method for false discovery rate (71). Lipid annotation for individual features was achieved by interrogation of accurate m/z values against the Mtb LipidDB database (41). Targeted features were quantified based on the calculated exact mass of known lipids using Skyline, an open-source software system (72) with the.mzML files.

#### Fas1 and Fas2 incorporation assays

To quantify Fas1-mediated *de novo* fatty acid synthesis the bacteria were grown in 7H12 butyrate at 37 °C, 6% CO_2_ in standing, vented T-25 flasks. Upon reaching an OD_600_ 0.25 to 0.4 the cultures were pulsed with 10 μCi of [1,2-^14^C] acetate in the presence of the indicated compounds for 24 h as described. The ^14^C-labeled bacterial cultures were pelleted and the cells were extracted with chloroform:methanol (2:1, v/v) three times. The pooled solvent extractable layers were dried to completion under a N_2_ bath. To quantify Fas2-mediated *de novo* mycolic acid synthesis the delipidated mAGP pellet remaining after the total lipid extraction was dried to completion under a N_2_ bath.

The ^14^C-labeled total extracted lipids and ^14^C-labeled mAGP fractions were suspended in 500 μl of chloroform:methanol (2:1, v/v) and 20% of each sample was quantified *via* liquid scintillation counting. For each fraction, the counts of vehicle control samples were considered 100% and all other samples were calculated as the percentage of ^14^C-incorporation in the treated samples relative to the control.

##### Growth assay to evaluate fitness of the HadC^E23K^ mutant

Mtb strains were pre-grown to mid-log phase in 7H12 acetate and used to inoculate 10 ml 7H9 OADC or 7H12 butyrate in vented T-25 tissue culture flasks to achieve an initial OD_600_ of 0.05. The flasks were incubated standing at 37 °C in a 6% CO_2_ environment and bacterial density at OD_600_ was measured every 3 to 4 days.

#### Mouse infections

Six-week old BALB/c mice (Jackson Laboratories) were infected with ∼200 colony-forming units of WT, HadC^E23K^, or HadC^E23K^ + pMV306(*hadABC*) using an aerosol inhalation exposure system (Glass-Col). After 7 weeks of infection, mice were sacrificed. Lung tissue was extracted and homogenized in PBS + 0.05% Tween-80 and plated on 7H10 OADC + cycloheximide plates for CFU enumeration. We confirmed that all three strains used in the mouse infections produce PDIM using metabolic labeling with ^14^C-propionate to track PDIM production. These animal studies were approved by the Institutional Animal Care and Use Committee at Cornell University (protocol number 2013-0030).

## Data availability

The data generated and analyzed in this study can be obtained from the authors upon reasonable request.

RNAseq metadata is publicly available (NCBI accession GCA_000350205.1).

## Supporting information

This article contains supporting information.

## Conflict of interest

The authors declare no conflicts of interest with the contents of this article.
