# Supplementary material for: Inhibition of de novo fatty acid synthesis in Mycobacterium tuberculosis
Source: J Biol Chem. 2025 Dec 8;302(1):111022. doi: 10.1016/j.jbc.2025.111022 (PMC12803843; doi:10.1016/j.jbc.2025.111022)
Supplement: Supporting Information [file mmc1.docx]

**Inhibition of *de novo* fatty acid synthesis in *Mycobacterium tuberculosis***

Emma K. Roszkowski^1^, Sarita Charap^1^, Christine R. Montague^1^, Paridhi Sukheja^2^, Case W. McNamara^2^, Paul S. Soma^3^, M Nurul Islam^5^, Barbara Graham^3^, Anna E. Grzegorzewicz^3^, Mary Jackson^3^, Baiyuan Yang^2^, Anthony G. Hay^6^, David G. Russell^1^, John T. Belisle^4^ and Brian C. VanderVen^1*^.

**Supporting information**

**List of material included:**

**Table S1 (separate Excel file)**

**Table S2**

**Table S3**

**Table S4 (separate Excel file)**

**Table S5 (separate Excel file)**

**Table S6 (separate Excel file)**

**Figure S1**

**Figure S2**

**Figure S3**

**Figure S4**

**Figure S5**

**Table S1 | RNAseq dataset.**

Provided separately as an Excel file

**Table S2. Frequency of colonies resulting from allelic replacement attempts to delete the native *hadABC* operon in strains that carry a functional *hadABC* operon integrated at the L5 site (WT-*hadABC*) or mutated variation of the hadABC operon integrated at the L5 site (MUT-*hadA^fs^BC^E23K^*).**

| **Strain** | **CFU** |
| --- | --- |
| WT*-hadABC* | 155 |
| MUT-*hadA^fs^BC^E23K^* | 6 |

**Table S3**. **Susceptibility of ISO-resistant mutants to sALT629.** EC_50_ measurments for *Mtb* mc^2^ 6206 WT and recombinant strains were performed in 7H9 + OADC + Tyloxopol + L-leucine + pantothenate + casamino-acid. EC_50_ values were determined by reading the size of the pellet and the reduction of resazurin.

| *Mtb* mc^2^ 6206 | EC_50_ sALT629 (µM) | EC _50_ ISO (µg/ml) |
| --- | --- | --- |
| WT | 12.5/25 | 5 |
| pVV16-HadABC | 50 | >20 |
| HadA^C61S^ | 6.25 | >20 |
| HadC^A151V^ | 25 | >20 |
| HadC^V85I^ | 6.25 | 5 |
| HadC^K157R^ | 50 | >20 |
| HadC^T123A^ | 25 | >20 |

**Table S4** | **Lipidomic dataset of total features detected in** **positive and negative mode.**

Provided separately as an Excel file

**Table S5** | **Lipidomic dataset of features with statistical differences in abundance levels relative to sALT629 treatment detected in** **positive and negative mode.**

Provided separately as an Excel file

**Table S6** | **Dataset of identified lipid features with statistical differences in abundance levels relative to sALT629 treatment detected in** **positive and negative mode**

Provided separately as an Excel file

**Figure S1**. **Differentially expressed genes in response to sALT629 and INH treatment**. **(A)** Venn diagram of upregulated genes in sALT629- and INH-treated cultures. **(B)** Heat map depicting the 50 most highly expressed genes following sALT629 and INH treatment. **(C)** Venn diagram of downregulated genes in sALT629- and INH-treated cultures. **(D)** Heat map of the 50 most downregulated genes following sALT629- and INH-treated cultures. Data are displayed as log_2_FC compared to the untreated control. Data are from three technical replicates from one experiment.

**Figure S2**. **Normalized abundances** **PCA of total features detected in positive and negative mode.** PCA of all statistically significant lipid features detected in positive **(A)** and negative **(B)** mode. PCA of the normalized abundances for all features demonstrated a clustering of the treatment groups with sALT629 forming its own cluster in both detection modes.

**Figure S3. Incorporation of ^13^C-acetate into phospholipid biosynthesis is inhibited by sALT629.** Total lipids were analyzed by LC-MS in negative ion mode to assess ^13^C-acetate incorporation in the presence of vehicle, sALT629 (25 μM), or INH (25 μM). **(A)** Ac1PIM2 (R1CO2H+ R2CO2H+R3CO2H+ = C51:0, R4 = H) [M-H]-. **(B)** PI (R1CO2H+ R2CO2H = C35:0) [M-H]-. **(C)** PE (R1CO2H+ R2CO2H = C35:0) [M-H]-.

**Figure S4**. **Loss of function** **HadC mutations increases sensitivity to antitubercular drugs**. Dose response curves of Erdman WT to **(A)** RIF and **(B)** bedaquiline (BDQ) in 7H12 butyrate media. Data are from two biological replicates with three technical replicates, each (n = 6). Error bars represent SD.

**Figure S5**. **Supplementation with pantothenate and free fatty acids fails to abrogate sALT629 killing**. **(A)** Dose-response curve of Erdman WT to sALT629 in 7H12 butyrate supplemented with 0.5% bovine-serum albumin (BSA) and 0 or 100 uM free fatty acid. **(B and C)** Dose-response curves of Erdman WT to sALT629 in 7H12 butyrate supplemented with **(B)** 0 or 1 mM pantothenate and **(C)** 0 or 100 uM biotin. Data are from two biological replicates with two technical replicates, each (n = 4). Error bars represent the SD.
